# Comparison of three qPCR-based commercial tests for detection of periodontal pathogens

**DOI:** 10.1038/s41598-021-85305-3

**Published:** 2021-03-17

**Authors:** Fridus Van der Weijden, Mirella Rijnen, Cees Valkenburg

**Affiliations:** 1 Clinic for Periodontology, Utrecht, The Netherlands; 2 Department of Periodontology, Academic Centre for Dentistry Amsterdam (ACTA), A Joint Venture Between the Faculty of Dentistry, University of Amsterdam and Faculty of Dentistry, Vrije Universiteit Amsterdam, Gustav Mahlerlaan 3004, 1081 LA Amsterdam, The Netherlands

**Keywords:** Microbiology, Risk factors

## Abstract

In periodontal practice microbial results of periodontal test kits for identification of key pathogens are an aid in the treatment planning. Information on the performance of commercially available test kits is therefore essential for the clinician. In this retrospective analysis three commercially available qPCR kits for detection and quantification of selected periodontal bacterial species were compared, using 100 clinical samples from patients with untreated periodontitis. The analysis involved two separate comparisons in which kit A (LabOral Diagnostics, The Netherlands) was compared with kit B (Advanced Dental Diagnostics, The Netherlands), and with kit C (OralDent diagnostics, The Netherlands). Analytic procedures for detection and quantification of selected periodontal bacterial species were carried out according to the instructions of the laboratories. Kit A detected target species more often, and absolute numbers of bacterial cells were higher than with kit B. A high degree of similarity was found between the test outcomes by kit A and kit C. *A*ll three kits performed satisfactory but small and significant differences exist between kits.

## Introduction

Periodontitis is a ubiquitous and irreversible biofilm initiated inflammatory condition^1^. The microorganisms in the dental biofilm are considered to be involved in the pathogenesis, and in particular the subgingival bacteria play an important role in initiation and progression^2^. The microenvironment of the subgingival pocket harbors a wide diversity of bacterial species^3^. Host factors contribute to the composition of dental plaque and to susceptibility to disease^4^. An imbalance in the total microbiota due to ecological stress results in an enrichment of some “oral pathogens” or disease-related micro-organisms. The World Workshop in Periodontics has provided a list of bacterial species that are associated with progression of periodontal attachment loss^5^. This selection of etiologically important bacterial species was based on prevalence in disease, immunological response, relevant virulence factors, experimental infections, and response upon treatment.

Potential pathogens associated with progression of disease include, among others, the strict anaerobic species *Porphyromonas gingivalis, Prevotella intermedia, Tannerella forsythia, Treponema denticola, Parvimonas micra, Fusobacterium nucleatum* and the capnophilic *Aggregatibacter actinomycetemcomitans*^5–9^. Additional bacteria that are difficult to grow have been identified as marker species of periodontitis based on DNA sequencing^10^. For example *Filifactor alocis*^7,11–13^ which has the potential to withstand oxidative stress^14^ and to induce strong pro-inflammatory responses^15^. Most suspected periodontal pathogens occur in microbial clusters some of which have been associated with disease progression. The most prominent cluster in this respect consists of the anaerobic and highly proteolytic species *P. gingivalis, T. forsythia* and *T. denticola,* and is often referred to as the red complex^16,17^. *P. gingivalis* has also been indicated as keystone pathogen because it can manipulate the native immune system of the host, by which it can facilitate its own survival and multiplication as well that as of the entire microbial community, and trigger inflammation^18^. The composition of the subgingival biofilm differs between individual patients. This can be the basis for a differentiated approach of periodontal therapy. In this respect, special attention has been paid to periodontal infections associated with *A. actinomycetemcomitans*, a species that has been implicated in periodontitis formerly classified as localized and generalized aggressive, and in refractory periodontitis^19,20^. Though the mere presence of *A. actinomycetemcomitans* cannot discriminate between different stages of periodontitis^22^. While pretreatment microbiological examination, especially for the detection of *A. actinomycetemcomitans*, was found to be a valuable screening method for identifying complex treatment need in adult patients with advanced periodontitis^23^. In patients harboring *A. actinomycetemcomitans* mechanical treatment does not predictably lead to resolution of the periodontal inflammation^21^.

Treatment of periodontitis is based on reduction of the total periodontal bacterial load, which is achieved by mechanical supra- and subgingival debridement. Open debridement can be achieved by periodontal surgery that provides direct access to the root for biofilm removal. Occasionally this therapy fails when periodontal attachment loss continues after treatment, or recurs after periods of remission^24^. This unsatisfying observation has led to the additional use of topical or systemic antibiotics in periodontal therapy^25,26^. The choice for systemic antimicrobial therapy is often based on clinical parameters but the use of microbiological diagnosis for decision making and choice of antibiotics has long been advocated^25,27,28^. This is based on the notion that differences in bacterial composition of the subgingival biofilm may require different treatment approaches, including the choice of adjuvant antibiotic therapy. This tailored approach connects with the medical aim to provide personalized medicine to the patient, and assist in prevention of under- and over treatment.

Different techniques have been applied to detect and quantify bacterial periodontal marker organisms such as bacterial cultivation, immunoassays, specific enzymatic tests and various molecular techniques. Commercially available test systems make use of DNA-chip technology, DNA-DNA hybridization, and qualitative and quantitative polymerase chain reaction (qPCR). Compared to bacterial culture, real time PCR provides a rapid diagnostic tool with which enables detection of small numbers of bacterial species in clinical specimens^29^. Untch and Schlagenhauf^30^ compared three different test systems using clinical periodontal samples, and concluded superiority in specificity and intra-test reproducibility of the qPCR technology. Comparison of three different qPCR test kits and using mock bacterial communities also revealed significant differences between the results^31^.

Multiple commercially available test kits based on qPCR are now available in Europe. For a dental care professional it is important to know that the performance of a qPCR test kit depends on the design of primers and probes, and may results in different outcomes between test systems. It is however difficult for the clinician to select which kit is most suitable without any clinically relevant data. The objective of the present practice based retrospective analysis was to compare three commercially available diagnostic qPCR kits for the detection and quantification of periodontal bacteria, using samples from patients diagnosed with moderate to severe adult periodontitis.

## Material and Methods

### Patients

Patients involved in the analysis had been referred because of periodontal problems by their general dentist to the specialist Clinic for Periodontology Utrecht, The Netherlands from 2013 up to 2016. The diagnostic procedure is described in an earlier paper^32^. In short, the clinical diagnosis was based on intra- and extra oral assessment, full mouth periodontal charting, and a relevant set of radiographs. The parameters that were collected included probing pocket depth (PPD; measurements were rounded off to the nearest millimeter), bleeding on pocket probing (BOP) scored as absent or present), and furcation involvement (using a PQ0W6 pocket probe and PQ2NM furcation probe, Hu-Friedy Inc.). PPD and BOP were recorded at six sites (mesio-buccal, buccal, disto-buccal, mesio-lingual, lingual and disto-lingual). Eligible patients met with the following criteria:

–no periodontal treatment in the past 6 months,

–no use of local or systemic antibiotics in the past three months,

–not pregnant,

–consenting to use the obtained data for research purposes.

### Design

Diagnostic microbiological testing is part of the standard treatment protocol in this clinic. The comparison of the outcome of three commercial microbiological test was initiated as an attempt for the clinic to decide which laboratory to work with. It consisted of two legs. In the first part, kit A used as benchmark^33^ was compared with kit B using a convenience sample of 50 clinical cases obtained from untreated periodontitis patients. In the second part of the analysis, kit A was compared with kit C using 50 clinical samples. The patient related data were retrospectively analyzed for the present paper.

### Test kits

Three commercially available qPCR test kits were used in this retrospective analysis: the Periodontal DNA Test (LabOral Diagnostics, Houten, The Netherlands, kit A), the Periodontal Bacteria Test (Advanced Dental Diagnostics B.V., Malden, The Netherlands, kit B), and the test system of Carpegen Perio Diagnostik (Carpegen GmbH, Münster, Germany, kit C), made available in the Netherlands by OralDent diagnostics. Each laboratory produced independent test results according to their own method of processing the microbial samples.

### Sampling

Subgingival biofilm samples were obtained using sterile endodontic paper points and a standardized sampling protocol^34^. Subgingival microbial specimens were taken from the site exhibiting the deepest probing depth, and showing bleeding on probing in each quadrant of the dentition^35–37^. The four sample sites were first isolated with cotton rolls, supragingival plaque was mechanically removed, after which two sterile paper points were inserted simultaneously to the depth of the periodontal pocket, and left in situ for approximately 10 s prior to removal. After extraction from the pocket one paper point from each of the four pockets was collected in a sterile vial of Kit A, and the other in a vial from either kit B or kit C. Samples were forwarded the same day under routine conditions by regular mail to each of the respective microbiology laboratories.

### Laboratory procedures

Samples were processed according to laboratory’s protocol. All three laboratory tested for the presence and numbers of *A. actinomycetemcomitans, P. gingivalis, P. intermedia, T. forsythia, P. micra, F. nucleatum,* and *T. denticola.* Test kit A is also equipped to detect and quantify *F. alocis.*

### Statistical analyses

The primary outcome was presence or absence of the test species. Secondary outcome was the number of bacterial cells of each detected species. The absolute numbers of bacterial cells of the test species in positive tested samples were log transformed (See online supplementary file 1). The means and standard deviations of all species were calculated as detected by the different test kits. The unpaired two-sided Student T-test was used to analyze differences in mean absolute numbers of bacterial cells. Sensitivity, specificity, together with 95% confidence intervals, % agreement, kappa values and McNemar Test χ^2^-test were calculated using R (4.03) via RStudio 1.3.1093^38^ and jamovi 1.07^39^. Bonferroni corrections for multiple comparisons were applied where appropriate. A p-value of < 0.05 was considered statistically significant.

### Ethical approval

All procedures performed in relation to the treatment of patients were in accordance with the ethical standards of the Clinic for Periodontology Utrecht and with the 1964 Helsinki declaration and its later amendments. Diagnostic microbiological testing is part of the standard protocol in this clinic. The study protocol (as registered under protocol number 202043) was reviewed by the Ethical Committee of the Academic Centre for Dentistry Amsterdam (ETC-ACTA) and judged to be exempt from the need for further ethical approval as it falls outside of the scope of the Medical Research Involving Human Subjects act (WMO). This study meets the ethical guidelines at ACTA (see online supplementary file 2).

### Informed consent

All data were procured retrospectively from the treatment records and entered anonymously in an Excel file. Patients had provided informed consent in advance that data related to their treatment could anonymously be used for further analysis.

## Results

In total 100 patients, referred to the Clinic for Periodontology Utrecht were sampled. In Table 1 the participants are ranked according to the Classification of Periodontitis^40^. Five patients were classified as stage II, 53 stage III and 42 stage IV. Of these 36 were graded as B and 64 as C. Table 2 shows the frequencies of periodontal pathogens detected by the kits A, B, and C. Table 3 presents the mean log number (standard deviation) of each bacterial species established in trial 1 and 2 by kit A, B, and C. Table 4 and 5 show the number of positive and negative samples for each species detected for kit A, kit B, and kit C.

**Table 1:** Staging and Grading^40^ of included participants (N = 100).

Stage & grade	N
II B	4
II C	1
III B	30
III C	23
IV B	2
IV C	40

**Table 2:** Number of samples (%) tested positive for selected periodontal pathogens by three different commercial qPCR test kits.

Species	Cohort 1 (N = 50, age 47.4, SD = 13.8)		Cohort 2 (N = 50, age 51.3, SD = 13.9)	
	Kit A	Kit B	Kit A	Kit C
*A. actinomycetemcomitans*	11 (22)	10 (20)	16 (32)	17 (34)
*P. gingivalis*	31 (62)	26 (52)	25 (50)	22 (44)
*P. intermedia*	31 (62)	11 (22)	32 (64)	30 (60)
*T. forsythia*	47 (94)	45 (90)	48 (96)	48 (96)
*P. micra*	48 (96)	44 (88)	50 (100)	50 (100)
*F. nucleatum*	50(100)	50 (100)	50 (100)	44 (88)
*T. denticola*	22 (44)	25 (50)	27 (54)	46 (92)
*F. alocis*	41 (82)	ND	43 (86)	ND

*ND* not analyzed by kit B and kit C.

**Table 3:** ^10^Log number of selected periodontal pathogens in samples tested positive by three different commercial qPCR test kits.

Species		Cohort 1 (n = 50)			Cohort 2 (n = 50)		
		Kit A	Kit B	P value	Kit A	Kit C	P value
*A. actinomycetemcomitans*		6.3 (1.0)	5.4 (1.2)	< 0.01	6.9 (1.5)	6.2 (0.83)	NS
*P. gingivalis*		6.9 (1.7)	5.5 (0.5)	< 0.001	7.2 (1.6)	6.7 (2.5)	NS
*P. intermedia*		5.8 (1.3)	3.2 (0.2)	< 0.001	3.8 (1.9)	3.7 (2.1)	NS
*T. forsythia*		6.3 (1.2)	5.3 (0.6)	< 0.001	5.9 (1.4)	6.2 (1.5)	NS
*P. micra*		4.4 (1.2)	3.2 (1.3)	< 0.001	4.6 (0.9)	5.9 (0.8)	P < 0.001
*F. nucleatum*		5.5 (0.9)	4.8 (0.7)	< 0.001	5.6 (0.6)	5.5 (1.7)	NS
*T. denticola*		5.5 (1.0)	5.6 (0.7)	0.60	5.9 (1.4)	6.2 (1.5)	NS
*F. alocis*		6.4 (0.93)	ND	NA	6.1 (0.9)	ND	NA

*ND* not determined, *NA* not applicable.

**Table 4:** Frequency of samples tested positive by two different test kits A and B.

	Aa	Kit B			Tf	Kit B		
Kit A		Present	Absent			Present	Absent	
	Present	10	1	11	Present	43	4	47
	Absent	0	39	39	Absent	2	1	3
		10	40	50		45	5	50

	Pg	Kit B			Pm	Kit B		
Kit A		Present	Absent			Present	Absent	
	Present	26	5	31	Present	43	5	48
	Absent	0	19	19	Absent	1	1	2
		26	24	50		44	6	50

	Pi	Kit B			Fn	Kit B		
Kit A		Present				Present	Absent	
	Present	11	20	31	Present	50	0	50
	Absent	0	19	19	Absent	0	0	
		11	39	50		50	0	50

	Td	Kit B						
Kit A		Present	Absent					
	Present	13	9	22				
	Absent	12	16	28				
		25	25	50

**Table 5:** Detection frequency of periodontal pathogens by two different test kits A and C.

	Aa		Kit C		Tf		Kit C	
Kit A		Present	Absent			Present	Absent	
	Present	16	0	16	Present	48	0	48
	Absent	1	33	34	Absent	0	2	2
		17	33	50		48	2	50

	Pg		Kit C		Pm		Kit C	
Kit A		Present	Absent			Present	Absent	
	Present	18	7	25	Present	50	0	50
	Absent	12	13	25	Absent	0	0	0
		30	20	50		50	0	50

	Pi		Kit C		Fn		Kit C	
Kit A		Present	Absent			Present	Absent	
	Present	30	2	32	Present	44	6	50
	Absent	0	18	18	Absent	0	0	0
		30	20	50		44	6	50

	Td		Kit C					
Kit A		Present	Absent					
	Present	26	1	27				
	Absent	19	4	23				
		45	5	50				

In trial 1, 50 consecutive clinical samples were analyzed with kit A and kit B. The number of positive samples of each of the test species were higher for all species with kit A except for *T. denticola .* A large difference was found in the frequency of samples that tested positive for *P. intermedia* (62% vs 22%). The majority (> 85%) of the samples gave positive results for *P. micra, F. nucleatum,* and *T. forsythia* whereas for other species a much lower positive outcome was observed *i.e. A. actinomytcetemcomitans, P. gingivalis, T. denticola (*Table 2*).* The number of bacterial cells per species detected was significantly higher with kit A as compared to kit B except for *T. denticola* positive samples (Table 3). Table 4 shows the number of positive and negative samples for each species detected by kit A and kit B. The mean number of *T. denticola* cells in positive tested samples was not different between kit A and kit B (log 5,48, SD 0.75 vs 5.59 SD 0.67, P = 0.06).

In trial 2 the frequencies of positive outcomes for the periodontal pathogens were rather similar except for *F. nucleatum,* which was higher with test kit A (100% vs 88%). *T. denticola* was found positive in lower frequency with test kit A (54% vs 92%) as compared to kit C.

*A. actinomycetemcomitans* emerged as positive outcome in approximately one third of the samples. With test kit C one additional sample was found positive. *P. gingivalis* was detected in approximately half of the samples with 3 additional positive samples by kit A. *P. intermedia* was detected in two additional samples by kit A. The mean log number of *A. actinomycetemcomitans*, *P. gingivalis, P. intermedia, T. forsythia, F. nucleatum,* and *P. micra* cells did not differ between kit A and kit C (Table 3). The mean log number of detected *T. denticola* cells was significantly higher with kit C compared to kit A (5.8 vs 2.7, P < 0.0001). Table 5 shows the number of positive and negative samples for each species detected by kit A and kit C.

In Table 6, data of the statistical analysis with respect to sensitivity, specificity, % agreement, and kappa values are presented. In the comparison of kit A versus B sensitivity varied between 36 and 100%. The χ^2^—test showed a significant difference with respect to the detection of *P. intermedia*. The % agreement associated with this was 60% and kapa value was 0.30. In the comparison of kit A versus C sensitivity varied between 88 and 100%. The χ^2^—test showed a significant difference with respect to the detection of *T. denticola* for which the specificity was 17%. The % agreement associated with this was 60% and kapa value was 0.15.

**Table 6:** Taking Test Kit A as the benchmark, sensitivity, specificity, together with 95% confidence intervals and % agreement and kappa values are computed for the presented microorganism in relation to either Kit B or Kit C.

Kit A versus Kit B	Sensitivity [95% CI]	Specificity [95% CI]	% agreement	Kappa	χ^2^-test
*A. actinomycetemcomitans*	91% [59–10]	100% [91–100]	98%	0.94	NS
*P. gingivalis*	84% [66–95]	100% [82–100]	90%	0.80	NS
*P. intermedia*	36% [19–55]	100% [82–100]	60%	0.30	0.007
*T. forsythia*	92 [80–98]	33% [1–91]	88%	0.19	NS
*P. micra*	90% [77–97]	50% [1–99]	88%	0.20	NS
*F. nucleatum*	100% [93–100]	anf	100%	anf	anf
*T. denticola*	59% [36–79]	57% [37–76]	58%	0.16	NS

Kit A versus Kit C	Sensitivity [95% CI]	Specificity [95% CI]	% agreement	Kappa	χ^2^-test
*A. actinomycetemcomitans*	100% [79–100]	97% [85–100]	98%	0.96	NS
*P. gingivalis*	88% [69–98]	100% [86–100]	94%	0.88	NS
*P. intermedia*	94% [79–99]	100% [82–100]	96%	0.92	NS
*T. forsythia*	100% [93–100]	100% [16–100]	96%	0.92	anf
*P. micra*	100% [93–100]	anf	100%	anf	anf
*F. nucleatum*	88% [76–95]	anf	88%	anf	anf
*T. denticola*	96% [81–100]	17% [5–39]	60%	0.15	0.007

Significant p-values for McNemar test χ^2^-test are given (Bonferroni correction for multiple comparisons) (*NS* not significant; *anf* analysis not feasible—due to the number of ‘zeros’ in the comparison).

Only test kit A was designed to detect and enumerate *F. alocis*. The frequency of positive test outcomes of this periodontal species was 82% and 86% in part 1 and 2 of this analysis (Table 2). The detected number of *F. alocis* was log 6.42 (SD 0.93) and 6.13 (SD 0.88) respectively (Table 3).

## Discussion

The microbiology of periodontal diseases has been the focus of intense investigation for several decades^41^. Various commercially available microbiological tests are available for the clinician to support in the diagnosis of periodontal disease^42^. Clinical findings ultimately determine the decision to treat, or retreat, for which the outcome of microbiological testing may provide guidance; e.g. whether or not to use antibiotics as adjuncts to mechanical therapy.

In the present retrospective analysis we compared three commercially available test kits for the detection and quantification of selected bacterial species associated with destructive periodontal disease. All three test systems are based on real time and quantitative polymerase chain reactions. Untch and Schlagenhauf^30^ compared DNA-DNA hybridization, semi-quantitative PCR, and qPCR using 20 samples from patients suffering from severe periodontitis. They showed superiority of the qPCR technique over hybridization and the semi-quantitative PCR, including the intra-test reproducibility. Their study results formed for the periodontal practice the basis for initiating the present comparisons of commercially available laboratory test. The analysis is the first to compare three qPCR kits using a relatively large number of clinical subgingival samples (N = 100). The most obvious differences were found between kit A and kit B. Both the prevalence of the selected species as well as the total mean number of bacterial cells per species was lower as compared to kit A. With kit A, a high prevalence of *F. alocis* was found in both trials. This species has been described as a strong biomarker for periodontitis due to its high subgingival detection in disease, and its absence in periodontal health^7,43^.

The data used for the present analysis were obtained from subgingival samples from patients that had been referred by their general dentist for periodontal disease. The level of disease was by the referring dentists considered to be beyond the control of what could be effectively treated in their general dental practice. Classification showed that these referrals were mainly Stage III and IV periodontitis patients with a fast majority being grade as C^40^. This provides a reflection of the population under investigation.

The information generated by microbiological analysis of subgingival plaque that has been collected from a periodontally diseased site, is highly dependent on the technique that is used for bacterial sampling. There are two primary methods by which a patient's subgingival plaque can be collected for subsequent analysis: removal using a curette or adsorption onto endodontic paper points^44^. Both require careful removal of supragingival plaque at the sampling site as was the case. The differences in frequency of positive outcomes are however not likely explained by the sampling technique since two endodontic paper points were entered simultaneously into the periodontal pockets before separating them for analysis.

The differences between kit A and C were less pronounced, with a slightly higher rate of positive outcomes for three species by kit A, for two species by kit B. Remarkable were the differences in frequency of *P. intermedia* and *T. denticola* by the three kits. For instance for *T. denticola* the highest frequency was observed by kit C (92%), and the lowest by kit A (44–54%). Differences in outcomes between kits may be explained by differences in DNA isolation, volume input in the PCR, the DNA multiplication program used and the composition of the mastermix. Also the use of nested PCR may influence test outcome, because this technique requires opening of the amplified DNA with risks of contamination, and increased reamplification of a specific amplicons from the first PCR amplification.

Microbiological diagnosis of destructive periodontal disease may aid in clinical decision making, and for the selection of adjuvant antimicrobial treatment^25^. The use of antibiotics has especially been advocated in the treatment of *A. actinomycetemcomitans*-associated aggressive periodontitis^27,45,46^ and *P. gingivalis/T. forsythia/ T.denticola*-associated periodontitis^47^. Therefore detection of these pathogens is essential in microbiological diagnosis. Relative to kit A, kit B did not detect *P. gingivalis* in four samples and in three samples by kit C. This indicates that further improvement and optimization of primer/probe sets is warranted. The reproducibility of the outcome of microbial analyses of periodontal samples between separate laboratories has been tested in the past using anaerobic culture technique, and was found to be rather poor^48^. A survey among European diagnostic oral microbiology laboratories demonstrated that the reason for this might be the lack of harmonization of laboratory processing methods^49^. The present analysis shows that the PCR technique has significantly improved the reproducibility between different laboratories.

Historically, inflammatory periodontal diseases have been recognized as being primarily of bacterial origin^50^. Contemporary microbiome studies indicate that individual pathogens are not always obvious etiologic agents in periodontitis/peri-implantitis. It is currently understood that these diseases manifest as a result of a disequilibrium in the dynamic relationships among biofilm, host, and microenvironment^51^. It should therefore be recognized that the results obtained from the analysis of the subgingival plaque do not provide sufficient information regarding the risk of disease progression. In this respect the absence of potential periodontal pathogens has been proposed as a better predictor for no further loss of attachment^52^. Clinical decisions should therefore never be made in isolation of clinical findings^44^. In the periodontal ecosystem diverse bacteria (or specific combinations of genes within the community) may be able to fulfill distinct roles that converge to form and stabilize a disease-provoking microbiota. Because the information gathered with microbiological tests is limited to a relatively small number of pathogens, current tests may fail to identify a keystone pathogen with a dysbiotic role for a particular patient. In the current context of elevated resistance to antibiotic medication there should however be a focus on microbiological diagnosis. This interest should inspire development of new and better laboratory techniques and interpretations, and ensure a successful and predictable treatment result for all types of periodontitis cases^27^. For instance a recent publication investigated the role of biologic markers in modelling periodontitis disease progression. The practical implication emerging from this research was that baseline biomarker expression profiles in biological fluids, and levels of *P. gingivalis* and *T. forsythia* in subgingival plaque, could predict outcomes associated with periodontal care prior to clinical detection^53^. Also with deep sequencing technologies novel periodontal disease-associated bacteria emerge^7,43,54^, one of which *(Candidatus Bacteroides Periocalifornicus)* was found in a recent study to have a strong association with the well-known pathogenic "red complex" that resides in deep periodontal pockets^54^.

The following limitations were recognized;

- With a lack of suitable information it was not possible to perform a sample size calculation ‘a priori’. Consequently a convenience sample of 50 was used, the number of samples which was considered to provide a clinically relevant outcome.
- It can not be ruled out that there were no differences in the microorganism being harvested by the two paper points even though they were inserted simultaneously.

In conclusion, the three commercially available qPCR test kits to support the microbiological diagnosis of periodontitis showed small but significant difference with respect to detection and quantification of selected bacterial species. Test kit A tested more frequently positive for *P. gingivalis* as compared to kit B and kit C and less frequently for *T. denticola*. Optimization of laboratory procedures and of primer/probe sets is needed to further improve the performance of all three test kits in order to provide a reliable reflection of actual presence and numbers of selected periodontal pathogens in the subgingival biofilm.

## Supplementary Information

Supplementary Information.
